# Cognitive control moderates parenting stress effects on children's diurnal cortisol

**DOI:** 10.1371/journal.pone.0191215

**Published:** 2018-01-12

**Authors:** Laurel Raffington, Florian Schmiedek, Christine Heim, Yee Lee Shing

**Affiliations:** 1 Berlin School of Mind and Brain, Humboldt-Universität zu Berlin, Berlin, Germany; 2 Center for Lifespan Psychology, Max Planck Institute for Human Development, Berlin, Germany; 3 Institute of Medical Psychology, Charité—Universitätsmedizin Berlin, Berlin, Germany; 4 German Institute for International Educational Research (DIPF), Frankfurt am Main, Germany; 5 Department of Biobehavioral Health, Pennsylvania State University, University Park, United States of America; 6 Division of Psychology, University of Stirling, Stirling, United Kingdom; 7 Department of Psychology, Goethe University Frankfurt, Frankfurt am Main, Germany; Southeast University Zhongda Hospital, CHINA

## Abstract

This study investigated associations between parenting stress in parents and self-reported stress in children with children's diurnal cortisol secretion and whether these associations are moderated by known stress-regulating capacities, namely child cognitive control. Salivary cortisol concentrations were assessed from awakening to evening on two weekend days from 53 6-to-7-year-old children. Children completed a cognitive control task and a self-report stress questionnaire with an experimenter, while parents completed a parenting stress inventory. Hierarchical, linear mixed effects models revealed that higher parenting stress was associated with overall reduced cortisol secretion in children, and this effect was moderated by cognitive control. Specifically, parenting stress was associated with reduced diurnal cortisol levels in children with lower cognitive control ability and not in children with higher cognitive control ability. There were no effects of self-reported stress in children on their cortisol secretion, presumably because 6-to-7-year-old children cannot yet self-report on stress experiences. Our results suggest that higher cognitive control skills may buffer the effects of parenting stress in parents on their children’s stress regulation in middle childhood. This could indicate that training cognitive control skills in early life could be a target to prevent stress-related disorders.

## Introduction

In middle childhood, parent-reported stress related to their parenting [1–3] and children’s self-reported impact of negative life events [4–6] have been shown to be viable indicators of children’s stress experiences. Research investigating the effects of stress in childhood has sought to better understand ‘how stress gets under the skin’ [7,8]. This is especially important in the developmental literature, because the developing brain is disproportionally more vulnerable to the adverse effects of stress than the adult brain [9]. Animal models have shown that glucocorticoid (GC) steroid hormones are causally affected by stress exposure, showing patterns of GC elevations or depression in response to stress, depending on the age of stressor exposure and presence of maternal care [10–13]. In humans, the endocrine stress response is mediated by the hypothalamus-pituitary-adrenal (HPA) axis, of which the GC cortisol is the end product. Cortisol secretion follows a diurnal rhythm with levels increasing starkly in the first hour upon awakening (cortisol awakening response; CAR) and decreasing linearly throughout the rest of the day (diurnal slope) [14]. Cortisol secretion mobilizes energy, suppresses the immune system, and helps the organism to adapt to stress [15], but comes at a cost with long-term activation [15]. Befitting cortisol secretion is necessary for optimal adaption to stress and both abnormally high and low cortisol levels have adverse effects on health [16]. Thus, HPA axis alterations in childhood and adolescence have been suggested to be a risk factor for later illness and psychiatric disorders although results have been somewhat inconsistent [6,17,18]. For instance, a recent meta-analysis found a significant association between flatter diurnal cortisol slopes and poorer health across multiple health domains [19].

A growing literature has linked environmental stressors to children’s cortisol secretion. Yet, commonly encountered stressors have been associated with both higher [20–22] and lower basal cortisol levels [23–27]. However, these studies have been methodologically limited by disregarding known diurnal secretory patterns in measuring a few serial ‘basal’ cortisol levels at differing times of the day. Furthermore, basal cortisol measures show considerably lower intra-individual stability than multiple measures of diurnal cortisol secretion collected across several days [28,29]. For example, confounding variables that influence cortisol levels, such as food intake or acute experiences of stress, affect basal cortisol levels more than multiple response measures. Recent recommendations have been made to improve collection and to control for confounds (see [30,31] for an expert consensus paper on measuring the CAR). Nevertheless, these basal cortisol secretion studies provide initial evidence for the notion that stressors affect children’s HPA axis functioning.

Measurement of more reliable dynamic cortisol secretion patterns has focused primarily on severe stress exposure, such as maltreatment [32,33] and early institutionalized care [34,35], finding *flattened* diurnal cortisol secretory patterns. One hypothesis is that flatter cortisol patterns may follow a phase of hyperreactivity of the HPA axis that leads to the down-regulation of the HPA axis to protect the developing system from overexposure to GC [12,36,37]. A small number of studies on less severe stress exposure indicate that higher parenting stress is associated with flatter diurnal cortisol profiles in 3-to-6-year-old children [38] and 9-to-12-year olds [39], but higher late morning cortisol levels in 3-to-5-year-old children [26]. Parents remain co-regulators of children’s cortisol stress responses to acute challenges into middle childhood [40], however stress renders parents less able to effectively co-regulate their child’s stress response [41,42] and affects parenting behaviors that can create stress for the child [1,2,43,44]. Lastly, parent-child interactions can in some cases elicit a cortisol stress response depending on the relationship quality [45]. Overall, these studies suggest that parenting stress in the parents is an important predictor of the child’s HPA axis function, although it is unclear in what way the diurnal cortisol secretion pattern is affected. Potentially, higher parenting stress could be associated with blunted diurnal cortisol secretion in children, mirroring effects of severe stress exposure.

Arguably a more valid measure of children’s stress experiences would be to evaluate children’s self-reported stress perception. The large majority of studies have focused on the effects of objective stressors (e.g., poverty) on stress physiology, disregarding the child’s subjective stress perception. Yet, stress occurs when an individual perceives an inability to cope with the demands of the environment [46]. The few studies to date suggest that higher self-reported stress perceptions in these multiple domains are associated with lower cortisol levels after awaking in 9-to-12-year-olds [4] and flatter diurnal cortisol slopes in 9-to-17-year-old children [5]. Furthermore, self-reported negative life events predicted the onset of depression in 9-to-14-year-old girls with higher levels of total cortisol levels [6]. Thus, daily stress perception may increase the risk for later psychiatric disorders by HPA axis dysregulation. In sum, it is not yet sufficiently established whether higher stress perceptions are associated with reductions or increases in diurnal cortisol secretion in childhood and whether higher or lower levels confer more health risk. Furthermore, the lack of correlation between parental and child stress reports may imply that young children are not reliable self-reporters of their stress experiences [47–50], in contrast to children over 9 years [4–6]. We know of no studies investigating self-reported stress and HPA axis activity focusing on middle childhood as a further test of the validity of using young children’s stress reports.

Importantly, not all children exposed to stress show HPA axis alterations [51]. This may be related to issues of reliability in basal cortisol measurements [29] or derive from moderation effects [51,52]. Psychological vulnerability and resilience factors are thought to play a vital modulatory role in the embedding of stress exposure [53]. For example, executive functions and prefrontal cortex (PFC) function have been suggested to protect against the development of behavioral problems and developmental disorders [54]. Additionally, a very large recent study suggests that high effortful control, low negative affect, and low emotional reactivity mitigated the negative associations between socioeconomic risk (marked by high stress level) and both reading and math development [55]. Therefore, executive functions may act as a moderator of the embedding of stress exposure, with high executive function abilities providing protective or resilience effects.

While no study to date has tested for executive function moderation effects on children’s cortisol secretion, previous research shows that behavioral self-regulation is correlated with cortisol secretion at daycare in early childhood [56–59]. For instance, preschool children with poorer self-control and more aggression showed greater increases in cortisol over the course of the day spent at daycare, suggesting that immature self-regulatory skills may stimulate cortisol elevations among young children [56]. Self-regulation is largely considered a cognitive regulatory skill and depends on executive functions including working memory, attention, and cognitive control, which can be defined here as top-down control of goal-directed action [60,61]. Indeed, the association of self-regulation and cortisol secretion was accounted for by an executive functions composite, including cognitive control [59]. Developmentally, cognitive control shows marked improvement in middle childhood [62,63] that intersects with functioning in multiple health, behavioral and physiological outcomes across the lifespan [64–67]. Furthermore, cognitive control abilities are reflected in PFC development [68], which is also critically involved in providing feedback control to the HPA axis in regulating cortisol secretion [69]. Therefore, we hypothesize that cognitive control may act as a moderator of the effects of stress exposure on HPA axis activity. This may partly explain mixed results associating cortisol levels with executive function that have found both higher [4,20,26,56,70] and lower cortisol levels [71–73] associated with outcomes of poorer executive functions.

To test this hypothesis, we investigated whether parenting stress and self-reported stress are associated with children’s diurnal cortisol secretion and whether this relationship is moderated by cognitive control capacity in middle childhood. First, we expected higher parenting stress and higher self-reported stress to be associated with flattened diurnal cortisol levels and a flatter diurnal slope, mirroring findings on previous diurnal cortisol studies and severe stress samples. Second, we predicted lower cognitive control to be associated with lower cortisol level. Thirdly, we expected higher cognitive control to buffer the negative effects of stress on diurnal cortisol secretion.

## Methods

### Participants

Participants were 53 children and their parents (29 female; age: *M* = 6.68; *SD* = .41) who took part in a longitudinal neuroimaging study (called the HippoKID study) that examined neurocognitive development with functional and structural magnetic resonance imaging. Data for the current study, other than the cognitive task, was collected only at the second time point of data collection (one year after baseline). Children were recruited at daycare centers and were either attending first grade or daycare during time point two testing. Strict health and behavioral exclusion criteria were specified, involving prenatal and perinatal histories, medical and psychiatric disorders, and history of steroid medication use. The study was approved by the Ethics Committee of the German Psychological Society (Deutsche Gesellschaft für Psychologie). All parents of participants provided written consent and children verbal assent.

### Procedure

Distributed across three sessions, computerized cognitive testing as well as scanning was completed individually in the presence of a trained experimenter at the laboratory. While participants were being tested, parents waited in a separate room and filled out demographic and parenting stress questionnaires. During session two, after cognitive testing, the experimenter read out items in the children’s self-report stress questionnaire and the child pointed to smiley faces corresponding to the response options. At the end of session two, parents were trained in collecting saliva samples from their children. Saliva samples were stored in parents’ home freezer and picked up by an experimenter within three weeks.

Parents were asked to collect samples on two weekend days to estimate stable trait-like profiles [29], since children tend to spend more time with their families on weekends thus maximizing potential effects of family and minimizing differences in daycare versus school attendance. Mean number of days between the collection days was 4.71 (SD = 6.45, range 1–28). Because the children were too young to collect their own samples, parents were asked to wake their child shortly before the time they would normally wake up to reduce the risk of prior awakening and take the saliva sample right away. Being woken-up has been shown not to affect the CAR in adults [74]. Following samples were taken 30 minutes after awakening, at 12:00, and right before dinner to minimize effects due to eating large meals [75]. The strict adherence to the study protocol, especially regarding the timing of sampling, was especially emphasized. Parents were trained to use a timer alarming them of sampling times. It was also stressed that saliva sampling had to be postponed to the next possible weekend day if the child had woken up spontaneously or fell ill. Parents were told to withhold food, drinks, and brushing teeth prior to the sample 30 minutes after awakening and to withhold meals and caffeine 2 hours prior to the 12:00 and pre-dinner samples. They were also asked to fill in a protocol recording sampling times and any problems.

Saliva samples were stored in parents’ home freezer and picked up by an experimenter within three weeks. They were then stored at -80°C until assayed. Samples were brought to room temperature and centrifuged at 3000 rpm for 15 minutes. All samples were assayed for cortisol concentrations at the Institute of Medical Psychology at Charité—Universitätsmedizin Berlin using a highly sensitive enzyme immunoassay (Salimetrics, Suffolk, UK). The test has a detection range from 0,012 μg/dL– 3 μg/dL with a lower sensitivity limit of 0,007 μg/dL and average intra- and inter-assay coefficients of variation were 6.64% and 4.19%, respectively. There were no cortisol samples below detection limit. All samples were assayed in duplicate and the average of duplicates was used in all analyses.

## Measures

### Cortisol levels

Cortisol collection and pre-processing steps followed recommendations made by an expert consensus as far as possible [31]. Time between first and second collections ranged from 20 to 40 minutes (M = 31.08, SD = 1.92). Given the rapid change in morning cortisol levels, this is not ideal even though every parent reported to be within a 15-minute compliance window [76]. Therefore, preliminary analyses were conducted to identify outliers in cortisol concentrations and values more than 4 SD from the mean and were ‘winsorized’, i.e. replaced with the value at the 99.7th percentile [77]. This affected six samples from Day 1 and two samples from Day 2. Missing samples (one from Day 1 and one from Day 2) were replaced by the mean of that sample. Because cortisol measures displayed skewness and kurtosis, a log transformation was applied to these concentrations after winsorizing to normalize their distributions and meet assumptions for statistical analyses. Cortisol values and collection times since awakening in minutes were mean-centered for statistical modeling. Raw cortisol values showed moderate correlations across time (see Table 1).

**Table 1:** Raw cortisol values, covariates and their correlations.

		Mean (SD)	1	2	3	4	5	6	7	8	9	10
1	Day 1 waking	0.31 (0.13)	1.00									
2	Day 1 waking +30	0.55 (0.19)	.28*	1.00								
3	Day 1 12:00	0.15 (0.07)	.10	.21	1.00							
4	Day 1 pre-dinner	0.09 (0.04)	.04	.21	.19	1.00						
5	Day 2 waking	0.29 (0.1)	.40*	.30*	.11	.02	1.00					
6	Day 2 waking +30	0.51 (0.15)	.24	.08	.09	.16	.37*	1.00				
7	Day 2 12:00	0.14 (0.06)	.15	.06	.35*	.29*	.28*	.34*	1.00			
8	Day 2 pre-dinner	0.09 (0.05)	.18	.11	.10	.44*	.23	.28*	.57*	1.00		
9	Week/ Weekend	20/79	.05	.11	-.03	.15	-.15	-.01	-.08	-.11	1.00	
10	Awakening time	7.23 (0.74)	-.21	-.19	0.22	.19	.04	-.11	.15	.16	-.04	1.00
11	Days between collection	5.74 (9.67)	-.14	-.07	-.02	-.03	-.01	.13	-.06	.16	-.18	.13

*Asterisks denote significant correlations at the *α* level of .05. Raw cortisol variables 1–8 are in **μg/dL.** Week/ Weekend day and Awakening time correlations use the corresponding day, so day 1 for correlations with cortisol 1–4 and day 2 for correlations 5–8.

### Parenting stress inventory

The Parenting Stress Inventory [78] is a widely used questionnaire that assesses stress as a consequence of parental role. Five subscales measure perceived stress due to child characteristics (Distractibility/Hyperactivity, Adaptability, Demandingness, Mood, Acceptability) and seven subscales measure parental characteristics and situational variables (Competence, Isolation, Attachment, Health, Role Restriction, Spouse/Parenting Partner Relationship, Depression). The validated German version of the questionnaire [79] was filled out by the parent who generally spent more time with the child and with the participating child in mind. Parents responded to 48 items on a 6-point scale ranging from strongly agree to (0) strongly disagree (5). One missing sample was mean replaced. The total score, which has good reliability (α = .91) [78], was divided by number of subscales (normally 12 subscales, but only 11 if the parent has no partner) and mean-centered for statistical modeling. Higher scores indicate increased parenting stress.

### Children’s stress questionnaire

The Children’s Stress Questionnaire has 50 items in five subscales and has been found to be reliable and valid in an Australian sample of children aged 7-to-9-years [80]. Although the full scale shows good reliability (α = .91), some of the subscales’ reliabilities are not so high (α < .60), suggesting that some caution may be needed using these scales [80]. Nevertheless, its validity has been established longitudinally by predicting scales of depression and anxiety [80]. Our analyses were restricted to the full scale. Five subscales measure pervasive hassles beyond normal control (every-day events such as not having enough money to buy things), relationship with parents (e.g., parents prefer siblings), experiences of transition (e.g., death of a family member), problems in school/daycare (e.g., bullying), and family dissonance (e.g., parental divorce). The questionnaire was translated into German by a Native German-English bilingual and then translated back into English by another Native German-English bilingual to highlight divergences. Disagreements were resolved through discussion. For each item, children pointed to the smiley corresponding to their self-reported impact, visualized as a crossed-out face (0 = ‘This did not happen to me’), neutral face (1 = ‘This happened, but did not affect me’), a very slight frown (2 = ‘This made me a little bit sad/angry’), a frown (3 = ‘This made me somewhat sad/angry’), a more exaggerated frown (4 = ‘This made me very sad/angry’) concerning their stressor experience over the past year. Raw scores were log transformed to correct for significant positive skew (*W* = 1.86, *p* < 0.05) and mean-centered for statistical modeling.

### Cognitive control

Cognitive control and flexibility was assessed using the Hearts and Flowers task [62]. On each trial, a heart (as the congruent stimulus) or a flower (as the incongruent stimulus) appeared on either the right or left side of a computer screen. The task started with a fixed order of blocks, starting from congruent, incongruent, to mixed blocks. In the congruent block, only trials with the heart stimulus were shown and the child was instructed to press the button on the same side as the heart. In the incongruent condition, only trials with the flower stimulus were shown and children were told to press the button opposite the flower. In the mixed block, trials with flower or heart were randomly shown, and the child was asked to press the congruent button when the heart appears and the opposite button when the flower appears. Each test block was preceded by a practice block, which was continued until the child achieved at least 62,5% correct. Stimuli were presented for 1500 ms, with an interstimulus interval of 500 ms. Incorrect responses or response latency less than 200 ms were excluded in the analysis. The variables of interest were mean accuracy and mean response latency on correct trials in the mixed condition divided by accuracy/latency on correct trials in the congruent condition. One missing sample was mean replaced. The latency outcome variable was multiplied by -1 so that a higher score indicated better cognitive control response speed and both variables were mean-centered for statistical modeling. See Table 2 for descriptive statistics of main variables and their correlation.

**Table 2:** Descriptive statistics of stress and cognitive control and their correlations.

		1	2	3	4
	Mean (SD)	9.12 (1.84)	23.96 (20.44)	1.90 (0.35)	0.85 (0.12)
1	Parenting Stress	1.00			
2	Self-reported Stress	.16	1.00		
3	Cognitive Control Speed	-.24	.04	1.00	
4	Cognitive Control Accuracy	.02	.01	.15	1.00

All *p*’s non-significant at the *α* level of .05.

### Data analysis

As the first step, a model of cortisol secretion was estimated using mixed effects hierarchical modeling, which has several strengths compared to more common repeated-measures ANOVA and difference scores reviewed elsewhere [31,81,82]. For example, the number of cortisol outcome variables and thereby the number of statistical comparisons is significantly reduced. In these hierarchical cortisol models [34,40,77], Level 1 represents repeated cortisol samples as a function of time, which are nested within subjects at Level 2. Our modeling was informed by previous studies that have shown that the combination of a linear time term (i.e., time since awakening), a quadratic time term (i.e., time since awakening–squared), and an imposed peak value at 30 min post-awakening provide good fit to diurnal cortisol data [77,83]. The linear time term represents the instantaneous rate of cortisol change and the quadratic time term represents the cortisol trajectory curvature. The peak term is a dummy variable indicating whether the value was the second sample or not to superimpose upon the quadratic model the typically observed peak elevation 30 minutes after awakening [77,83]. The intercept, linear time, and quadratic time terms were entered as random effects consecutively and retained if they improved model fit. Entering linear time, quadratic time, and peak variables without interactions best fit the data and adding random effects for intercept and linear time terms and their covariance significantly improved fit. Those random effects capture between-person differences in the overall cortisol level (intercept term) and cortisol trajectory over time.

In a second step, we explored whether potential covariates significantly predicted cortisol secretion by adding them consecutively to the model and testing for interactions with time. None of the covariates (age, gender, awakening times, week/weekend day, number of days between samples, school versus daycare attendance) showed significant main effects or interactions with time variables.

In a third step, we added stress variables (parenting stress and self-reported stress in separate models) as main fixed effects, and explored their interactions with time variables, only retaining significant effects. We finally added cognitive control, separately for response speed and accuracy, exploring both main effects and interactions with time and stress variables.

Models were implemented using the lme4 package [84] for linear mixed modeling in R and compared via likelihood ratio tests. Significance (at the α level of .05) of individual parameter estimates were evaluated by comparing the log-likelihoods of the full model and models leaving out the corresponding effect using χ^2^ tests. Significant effects were bootstrapped with 5000 iterations and we report bootstrapped confidence intervals (CI). Specific values for the parameter estimates were used to follow up on significant interactions and interpret directionality of effects through simple slope analysis [85].

Finally, to aid comparability to other studies and following recommendations by Stalder and colleagues [31], we further describe effects of stress on the CAR and diurnal slope by running linear regression models on the first cortisol level at awakening, the mean morning increase (0–30 min delta), and the diurnal slope (30 min–evening delta). These models averaged outcome variables across the two days and included awakening time and time since awakening as covariates.

## Results

### Cognitive control response speed

**Fig 1:**
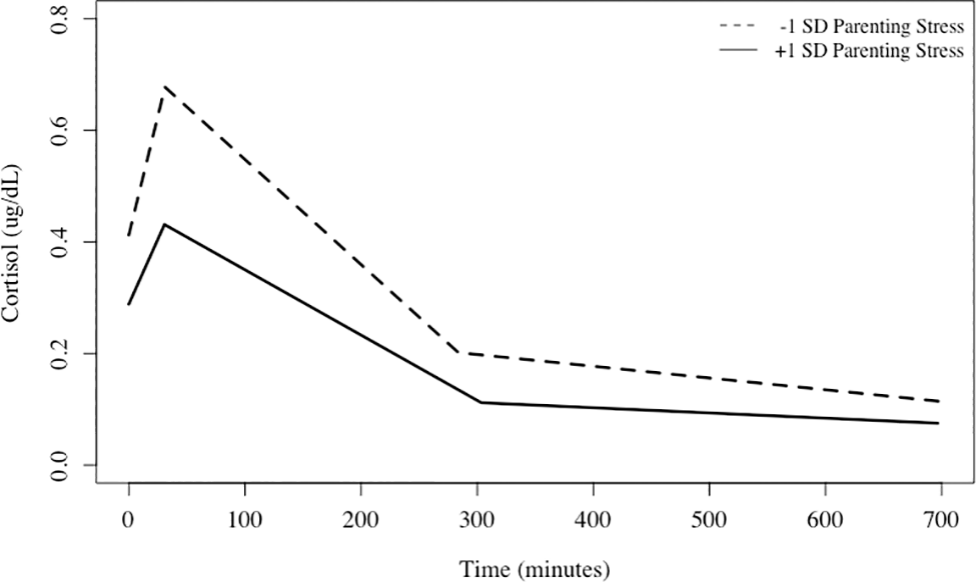
Diurnal cortisol levels in children with parents of high or low parenting stress. Mean raw diurnal cortisol levels in children of parents with high (+1 SD) and low (-1 SD) parenting stress. Actual models included parenting stress as a continuous variable. Higher parenting stress was associated with lower total cortisol levels over the day and there were no significant time-sensitive effects.

**Fig 2:**
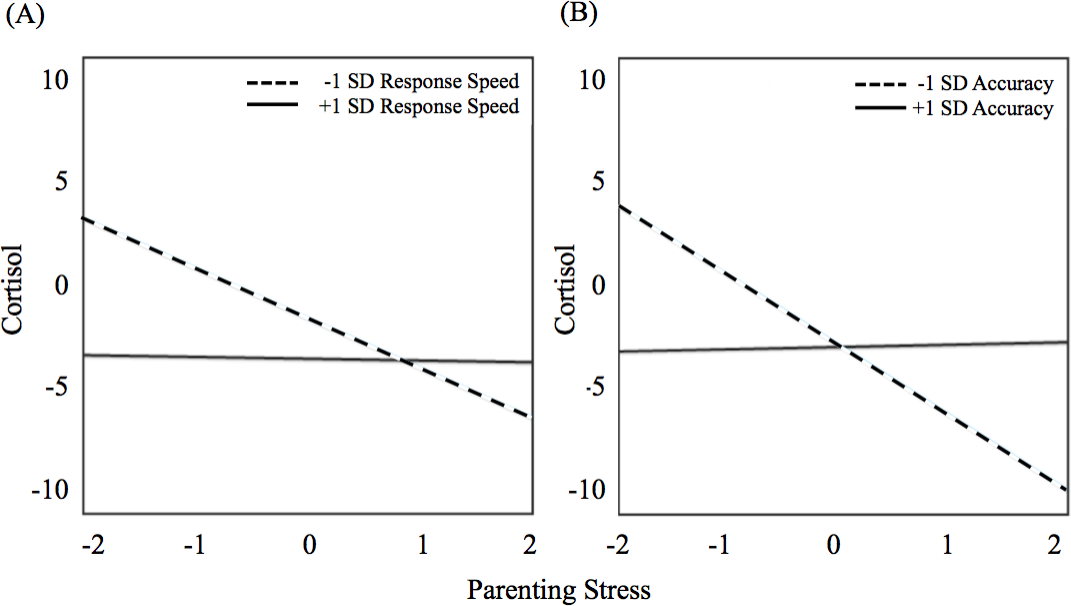
Cognitive control moderates parenting stress effects on diurnal cortisol levels. Relationship between parenting stress and diurnal cortisol levels in children with high and low cognitive control, indicated by response speed (A) and accuracy (B).

We first tested whether parenting stress and cognitive control speed, as well as their interaction, was related to cortisol secretion in hierarchical models (see Tables 1 and 2 for descriptive statistics of main variables of interest and their correlations). Confirming the first hypothesis, the negative main effect of parenting stress on cortisol secretion was significant, suggesting reduced diurnal cortisol level with higher parenting stress (see Fig 1; see Table 3 for parameter estimates). There was a significant negative main effect of cognitive control response speed, which contradicts the second hypothesis. There were no significant interactions of stress or cognitive control variables with linear, quadratic, or peak time effects. However, there was a significant parenting stress x cognitive control response speed interaction (see Fig 2A). For children with lower cognitive control, higher parenting stress was related to reduced diurnal cortisol level (Simple slope at Z = -1: -2.19, *t*(50) = 3.61, *p* < 0.05), whereas for children with higher cognitive control, parenting stress was not related to cortisol level (Simple slope at Z = 1: -0.08, *t*(50) = 0.11, *p* = 0.91).

**Table 3:** Parameter estimates and model indices for cortisol secretion with parenting stress and cognitive control response speed as predictors.

**Level 1**				
**Random Effect**	**Variance**	**SD**		**Δχ**^**2**^
Intercept	6.66	2.58		47.54
Linear time	2.06	1.44		14.70
Residual	21.45	4.63		–
**Fixed Effects**	**β**	**SE**_**β**_	**95% CI**	**Δχ**^**2**^**(1)**
Linear time	-7.58*	0.42	-8.38 –-6.70	132.83*
Quadratic time	1.25*	0.35	0.31–1.92	12.50*
Peak	5.75*	0.60	4.49–6.98	82.26*
**Level 2**				
**Fixed Effects**	**β**	**SE**_**β**_	**95% CI**	**Δχ**^**2**^**(1)**
Parenting stress	-1.02*	0.45	-1.63 –-0.63	5.36*
Cognitive control response speed	-0.81*	0.40	-1.86 –-0.43	4.05*
Parenting stress x Cognitive control response speed	1.15*	0.51	0.65–2.00	5.20*

*Asterisks denote significance at the *α* level of 0.05. The Δ*χ*^*2*^ values refer to likelihood ratio tests with one df resulting from model comparisons of the full model with a model leaving out the corresponding effect.

Next, we examined whether self-reported stress and cognitive control speed, as well as their interaction, was related to cortisol secretion in hierarchical models. Contrary to our hypothesis, self-reported stress was not related to cortisol secretion. There were no significant interactions of self-reported stress with linear, quadratic, or peak time effects and no significant self-reported stress x cognitive control response speed interaction (see Table 4).

**Table 4:** Parameter estimates and model indices for cortisol secretion with self-reported stress and cognitive control response speed as predictors.

**Level 1**				
**Random Effect**	**Variance**	**SD**		**χ**^**2**^
Intercept	8.96	2.99		67.60*
Linear time	2.05	1.43		15.71
Residual	21.45	4.63		–
**Fixed Effects**	**β**	**SE**_**β**_	**95% CI**	**Δχ**^**2**^**(1)**
Linear time	-7.57*	0.42	-8.40 –-6.67	132.85*
Quadratic time	1.24*	0.35	0.24–1.92	12.25*
Peak	5.75*	0.60	4.51–6.98	82.23*
**Level 2**				
**Fixed Effects**	**β**	**SE**_**β**_	**95% CI**	**Δχ**^**2**^**(1)**
Self-reported stress	0.33	0.43	-0.16–1.04	0.43
Cognitive control response speed	-0.40	0.43	-1.40 –-0.05	0.35
Self-reported stress x Cognitive control response speed	0.28	0.46	-0.43–1.17	0.39

*Asterisks denote significance at the *α* level of .05. The Δ*χ*^*2*^ values refer to likelihood ratio tests with one df resulting from model comparisons of the full model with a model leaving out the corresponding effect.

### Cognitive control accuracy

Subsequently, we tested whether parenting stress and cognitive control accuracy, as well as their interaction, was related to cortisol secretion in hierarchical models. Again contradicting the second hypothesis, there was no main effect of cognitive control accuracy on cortisol levels (see Table 5 for parameter estimates). There were no significant interactions of stress or cognitive control variables with linear, quadratic, or peak time effects. However, as in the response speed model, there was a significant parenting stress x cognitive control accuracy interaction (see Fig 2B). For children with lower cognitive control accuracy, higher parenting stress was related to reduced diurnal cortisol levels (Simple slope at Z = -1: -3.12, *t*(50) = 2.81, *p* < 0.05), whereas for children with higher cognitive control, parenting stress was not related to cortisol levels (Simple slope at Z = 1: 0.10, *t*(50) = 0.15, *p* = 0.88).

**Table 5:** Parameter estimates and model indices for cortisol secretion with parenting stress and cognitive control accuracy as predictor.

**Level 1**				
**Random Effect**	**Variance**	**SD**		**Δχ**^**2**^
Intercept	8.11	2.84		65.10
Linear time	2.03	1.43		19.38
Residual	21.46	4.63		–
**Fixed Effects**	**β**	**SE**_**β**_	**95% CI**	**Δχ**^**2**^**(1)**
Linear time	-7.57*	0.42	-8.29 –-6.59	133.02*
Quadratic time	1.24*	0.35	0.15–1.83	12.25*
Peak	5.74*	0.60	4.55–6.96	82.27*
**Level 2**				
**Fixed Effects**	**β**	**SE**_**β**_	**95% CI**	**Δχ**^**2**^**(1)**
Parenting stress	-1.46*	0.50	-2.02 –-0.91	8.41*
Cognitive control response accuracy	-0.09	0.43	-0.85–0.49	0.83
Parenting stress x Cognitive control response accuracy	1.72*	0.81	0.63–2.37	4.64*

*Asterisks denote significance at the *α* level of .05. The Δ*χ*^*2*^ values refer to likelihood ratio tests with one df resulting from model comparisons of the full model with a model leaving out the corresponding effect.

Next, we examined whether self-reported stress and cognitive control accuracy, as well as their interaction, was related to cortisol secretion in hierarchical models. Contrary to our hypothesis and mirroring speed results, self-reported stress was not related to cortisol secretion. There were no significant interactions of self-reported stress with linear, quadratic, or peak time effects and no significant self-reported stress x cognitive control response accuracy interaction (see Table 6).

**Table 6:** Parameter estimates and model indices for cortisol secretion with self-reported stress and cognitive control response accuracy as predictors.

**Level 1**				
**Random Effect**	**Variance**	**SD**		**Δχ**^**2**^
Intercept	9.50	3.08		74.04*
Linear time	2.04	1.43		16.98*
Residual	21.46	4.63		–
**Fixed Effects**	**β**	**SE**_**β**_	**95% CI**	**Δχ**^**2**^**(1)**
Linear time	-7.57*	0.42	-8.34 –-6.63	132.89*
Quadratic time	1.24*	0.35	0.16–1.90	12.21*
Peak	5.75*	0.60	4.51–6.99	82.22*
**Level 2**				
**Fixed Effects**	**β**	**SE**_**β**_	**95% CI**	**Δχ**^**2**^**(1)**
Self-reported stress	0.25	0.44	-0.32–0.83	0.34
Cognitive control response accuracy	-0.13	0.53	-1.12–0.50	0.06
Self-reported stress x Cognitive control response accuracy	0.11	0.66	-0.46–1.35	0.86

*Asterisks denote significance at the *α* level of .05. The Δ*χ*^*2*^ values refer to likelihood ratio tests with one df resulting from model comparisons of the full model with a model leaving out the corresponding effect.

In addition, we further investigated effects of parenting stress on diurnal cortisol secretion using more common CAR and diurnal slope indices in linear regression models to aid comparability to other studies. Parenting stress was associated with a significantly lower cortisol level at awakening (β = -0.04, SE = 0.02, *CI* = -0.09 - -0.01, *p* < 0.05), but not mean morning increase (0–30 min delta) (β = -0.03, SE = 0.02, *CI* = -0.08–0.01 *p* = 0.17). Further, parenting stress was associated with a flatter diurnal slope (β = 0.07, SE = 0.02, *CI* = 0.02–0.12, *p* < 0.05). However, this was reduced to a non-significant trend (β = -0.04, SE = 0.02, *CI* = -0.01–0.10, *p* = 0.07), when including the significant effect of cortisol level at awakening (β = -0.05, SE = 0.17, *CI* = -0.80 –-0.10, *p* < 0.05) [31]. Therefore, the conservative interpretation of our data derived from converging these regression results with the lack of time-sensitive effects in the hierarchical model is that parenting stress is associated with total cortisol levels starting with lower morning cortisol levels that persist to be lower over the rest of the day, but is not significantly associated with the CAR or diurnal slopes.

## Discussion

This study investigated associations between parenting stress and self-reported stress with children's diurnal cortisol secretion, and whether this is moderated by children’s cognitive control. Partially confirming our first hypothesis, higher parenting stress, but not self-reported stress, was associated with lower diurnal cortisol levels. Additionally, we found that cognitive control moderated this relationship, such that higher parenting stress was associated with reduced diurnal cortisol levels only for children with lower cognitive control.

First, our finding that prepubescent children’s cortisol secretion under parenting stress is profiled by a reduction in total diurnal cortisol secretion is in line with other studies finding total output reductions and flatter diurnal slopes [5,38,39,86], although we observed no reliable time-sensitive differences in CAR or diurnal slope. We speculate that we did not replicate time-sensitive slope differences over the day, because our sample had limited stress variance and a moderate sample size. Nevertheless, relative to a large norming sample of German mothers [79], parenting stress in our study ranged from the <2 to <98^th^ percentile with the mean falling into the 62^nd^ percentile. Since total cortisol levels has been shown to have the highest level of stability compared to the CAR and diurnal slope [29,87], this may explain why parenting stress is most reliably associated with total output in our study. However, another study found a positive association with higher late morning cortisol levels in 3-to-5-year-old children being related to higher parenting stress [26]. Critically, these children were younger and morning cortisol secretion was measured at daycare, where the parents are not present and parental presence seems to moderate cortisol secretion in young children and rodents [12,40]. Therefore, the null effect of parenting stress on CAR and diurnal slope should be interpreted cautiously. Future studies should measure diurnal cortisol secretion in children and adolescents on 4 days to enhance reliability including bedtime levels [29].

The reduction of diurnal cortisol secretion we and others have found in middle and later childhood is in line with patterns seen in children living in severe instances of stress, such as maltreatment [32,88]. The mechanisms involved in reduced diurnal cortisol secretion are not clear. Potentially, low cortisol levels are the result of sustained increases in cortisol secretion in response to chronic stress that result in flattened diurnal rhythm to reduce neural damage compensating for overexposure to cortisol [12,15,16,89]. It remains to be established whether cumulative chronic stress and heightened chronic cortisol levels [90–93] precede flattened diurnal cortisol secretion in more stressed children, a chronic stress mechanism that has garnered some support in mediating higher and lower basal cortisol levels [94,95]. Especially aspects of early childhood caregiving may be operative in shaping HPA axis activity in middle childhood [96], which may further explain findings of higher cortisol levels in younger children [26].

Contrary to our first hypothesis and unlike previous studies, we found no effects of self-reported stress of children on cortisol secretion [4–6]. One major difference between these studies and the present study is that the children in our sample were several years younger and may not yet be reliable reporters of their stress experiences. Self-reported stress was on the lower end of the scale—the mean was approximately 20 points lower than the 7-9-year-old Australian sample with which the questionnaire was devised [80]—and did not correlate with parenting stress similar to previous studies [47–50]. Given the positive skew in the scale, this indicates that 6-to-7-year-old children cannot report their stress experiences or that there is little variance in stress perceptions. However, even children aged 7–15 years have reported lower perceived stress compared to adults in response to the same acute stress task despite comparable cortisol reactivity levels [97]. Thus, the association of stress perception on diurnal cortisol secretion in childhood is likely to be dependent on age and should be distinguished from parental reports.

Second, the association of cognitive control and cortisol secretion was inconsistent. While lower cognitive control response speed was associated with higher, not lower, cortisol, cognitive control accuracy was not associated with cortisol at all. This mirrors the mixed literature with some studies suggesting a negative cortisol and executive functions association [4,20,26,56,70] and other studies finding a positive association, as well as null associations within these studies [26]. Therefore, the literature on executive functions and cortisol is mixed. Since not all children exposed to stress show HPA axis alterations or develop cognitive deficits, these mixed results may in part stem from lacking a consideration of moderation effects.

In support of this notion and confirming the third hypothesis, we found that cognitive control modified the relationship between parenting stress and diurnal cortisol. In particular, higher parenting stress was associated with reduced diurnal cortisol output in children with lower cognitive control, but not in children with higher cognitive control. Our results suggest that higher cognitive control skills may buffer the effects of parenting stress on their children’s stress physiology in middle childhood. Presumably, higher cognitive control skills are related to higher emotional self-regulation [59], which enables these children to ward off stress responses otherwise transferred by their parents. These finding suggests that executive functions need to also be considered as moderators, not just outcomes, of stress physiology in the developmental stress literature. Even more so, if cognitive control is both a moderator and outcome of chronic stress exposure, this could indicate a snowball effect of psychological vulnerability leading to worse cognition, such that worse cognitive control facilitates the embedding of chronic stress, leading to worse cognitive control and so on. Cross-lagged longitudinal studies could test for such bidirectional dynamics. Given the role of HPA axis in health, these results have larger implications for the risk of psychiatric and health disorders [6]. Therefore, investigating psychological vulnerability and resilience factors to stress is an important area of ongoing research [53]. An important challenge will be to relate such cognitive moderators to genetic effects, such as those hypothesized to make some individuals more susceptible to their environments.

This study has several limitations. The cross-sectional nature of the data did not allow for cause-effect inferences to be made concerning parenting stress affecting child diurnal cortisol secretion/levels or vice versa. It is also possible that children with lower diurnal cortisol and cognitive control may increase their parents’ stress, because of behavioral difficulties; or that those children share genes with their parents that reduces diurnal cortisol secretion, making them both more stress prone. While we know of no longitudinal HPA axis studies investigating bidirectional effects with parenting stress, a related longitudinal study found that child emotion regulation and externalizing behaviors predicted parenting stress longitudinally at 2, 4, and 5 years [98]. Thus, bidirectional influences of children’s emotion regulation, HPA axis and parenting are plausible. Alternatively, both higher parenting stress and lower diurnal cortisol could derive from genetic similarities between children and their parents that influence stress reactivity. More longitudinal research is needed to understand the lead-lag interplay of children’s HPA axis functioning and their parents’ stress, preferably in consideration of heritability.

Second, small variations in timing can lead to large differences in cortisol levels, which is why the use of objective measures of awakening times using movement trackers and sampling times using electronic time monitoring devices have been recommended for use, but were not available at the present data collection [31,99]. Finally, this study focused on healthy children in a rather small sample and excluded children with diagnosed psychiatric disorders; hence our results are not readily generalizable to wider populations and possibly revealed only the lower bound of associations of stress and HPA axis functioning.

In conclusion, our study showed that higher parenting stress was associated with lower diurnal cortisol output in 6-to-7-year-old children. This effect was moderated by cognitive control, suggesting that children with lower cognitive control had reduced diurnal cortisol output under high parenting stress, whereas children with higher cognitive control showed no associations of cortisol and parenting stress. There were no effects of self-reported stress on cortisol secretion. In conclusion, cognitive control is an important individual difference characteristic to be considered in future studies that potentially modulates the effects of parenting stress on child’s stress physiology. Importantly, executive function and self-regulation is modifiable to interventions, especially in at-risk children, thus training these skills could reduce the occurrence of stress-related disorders.

## Supporting information

S1 Data Anonymized raw data text file of variables of interest. (TXT)

S2 Data Variable key and description of raw data text file S1 Data. (TXT)
